# Supplementary material for: Effectiveness of Simulation with a Standardized Patient on Knowledge Acquisition, Knowledge Retention, and Self-Efficacy Among Moroccan Nursing Students: A Quasi-Experimental Study
Source: Healthcare (Basel). 2025 Feb 4;13(3):318. doi: 10.3390/healthcare13030318 (PMC11816925; doi:10.3390/healthcare13030318)
Supplement: Supplementary file 1 [file healthcare-13-00318-s001.zip › healthcare-3308489-supplementary.pdf]

## Supplementary Materials: Table S1: Pedagogical scenario

### Patient Admitted to the Emergency Department with Hemorrhagic Shock

**General Objective:** To systematically apply the nursing process when managing a patient in hemorrhagic shock.

| Situation Presented to Students | Objectives for Students | Expected Actions |
| --- | --- | --- |
| A 47-year-old patient is admitted to the emergency department after a high fall, presenting with a significant open wound on the left leg with active bleeding. The patient exhibits signs of hemorrhagic shock, including severe hypotension (85/50 mmHg), rapid and thready tachycardia (125 bpm), pallor, cold sweats, prolonged capillary refill time (>3 seconds), and mild confusion. | - Quickly recognize the clinical signs of hemorrhagic shock.<br>- Initiate appropriate emergency interventions to control bleeding and stabilize hemodynamic status.<br>- Ensure adequate tissue oxygenation.<br>- Continuously assess vital signs and monitor the clinical condition.<br>- Effectively coordinate care with the emergency medical team. | - Apply direct pressure to the wound to stop active bleeding.<br>- Prepare and, if necessary, use a tourniquet to control persistent hemorrhage.<br>- Initiate rapid intravenous infusion of crystalloids through two peripheral access points.<br>- Prepare for blood transfusion, if necessary, based on the severity of the hemorrhage.<br>- Administer high-flow oxygen (15 L/min) via a high-concentration mask to improve tissue oxygenation.<br>- Continuously monitor vital signs (BP, HR, RR, $SpO_2$) and look for signs of decompensation.<br>- Maintain effective communication with the medical team to coordinate care and organize rapid transfer to an intensive care unit or operating room.<br>- Document all interventions performed (compression, infusions, oxygen therapy, vital signs) with time stamps and evaluate the effectiveness of the actions taken. |

**Figure S1: Hemorrhagic shock management algorithm**

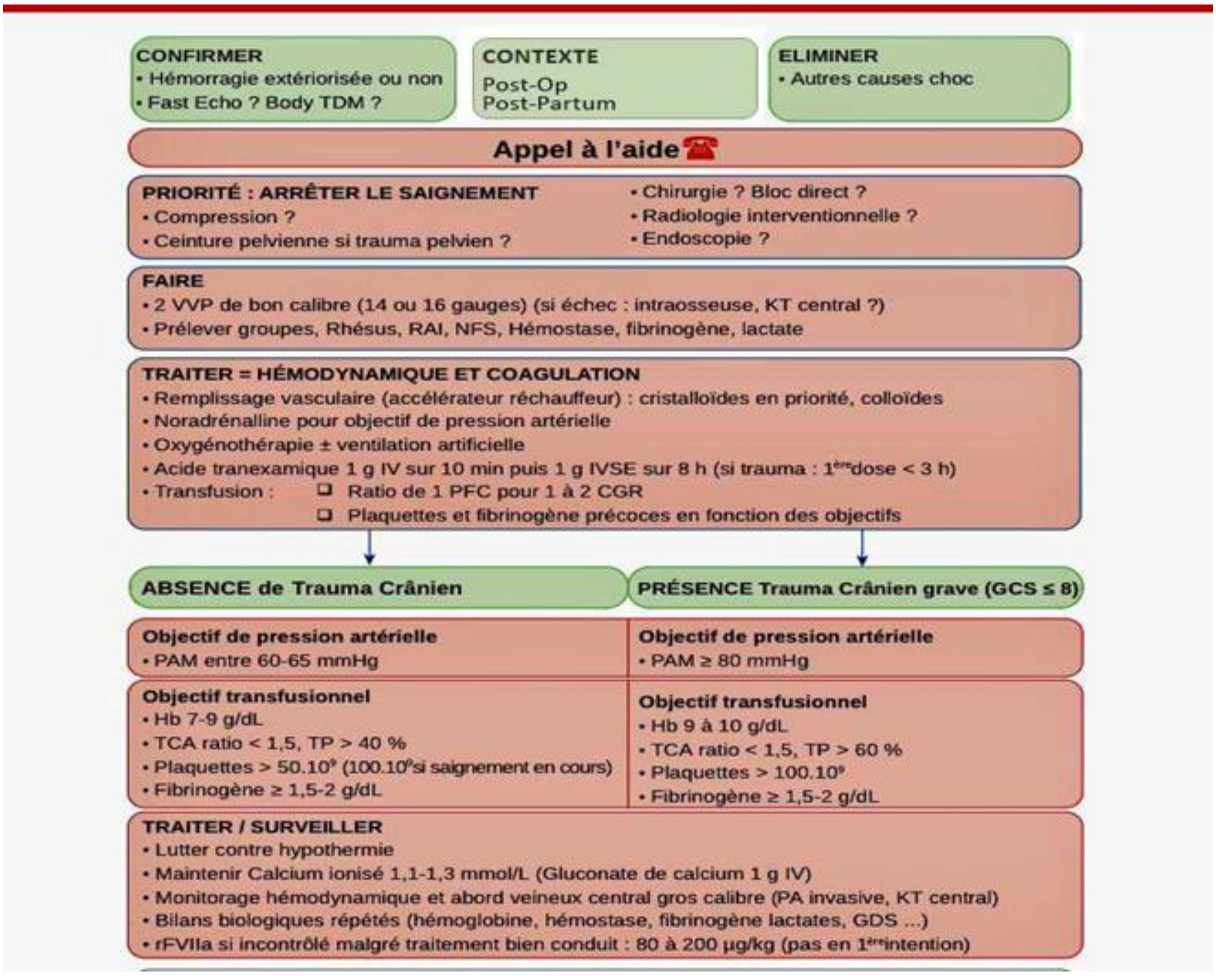

## Supplementary File S1: Knowledge Test

1. **What is one of the first clinical signs of hemorrhagic shock?**
   a) High fever
   b) Low blood pressure
   c) Hyperglycemia
   d) Cyanosis

2. **What clinical sign is typical of hemorrhagic shock in a conscious patient?**
   a) Bradycardia
   b) Tachypnea
   c) Blurred vision
   d) Vomiting

3. **Hemorrhagic shock can manifest with skin that is:**
   a) Cold and clammy
   b) Red and warm
   c) Dry and pale
   d) Normal without changes

4. **Which biological parameter may indicate hemorrhagic shock?**
   a) Hyperkalemia
   b) Decrease in hemoglobin
   c) Increase in white blood cells
   d) Hypercalcemia

5. **What is the most frequently monitored vital parameter to detect a state of shock?**
   a) Respiratory rate
   b) Heart rate
   c) Mean arterial pressure
   d) Body temperature

6. **What method is used to continuously monitor oxygen saturation?**
   a) Capnography
   b) Pulse oximetry
   c) Arterial blood gas
   d) Electrocardiogram

7. **During clinical monitoring of a patient in hemorrhagic shock, which sign indicates rapid deterioration of the clinical condition?**
   a) Increased urine output
   b) Persistent tachycardia
   c) Oxygen saturation of 98%
   d) Normal blood pressure

8. **How frequently should vital signs be assessed in a patient with hemorrhagic shock?**
   a) Every 4 hours
   b) Every 2 hours
   c) Every 15 to 30 minutes
   d) Once a day

9. **What is the first action to control external bleeding?**
   a) Apply a tourniquet immediately
   b) Apply direct pressure to the wound
   c) Initiate vascular filling
   d) Immobilization

10. **What is the priority intervention to stabilize hemodynamic status in a patient with hemorrhagic shock?**
a) Administer anticoagulants
b) Place the patient in a seated position
c) Initiate vascular filling
d) Provide analgesia and sedation

11. **When should a tourniquet be applied to control bleeding?**
a) In the case of minor bleeding
b) When direct compression is ineffective
c) For all deep wounds
d) After a blood transfusion

12. **What is the correct approach to assess the need for a blood transfusion in a patient with hemorrhagic shock?**
a) Assess only vital signs
b) Quickly measure hemoglobin and hematocrit levels
c) Wait for the patient to show signs of cardiac arrest
d) Observe the color of the skin and mucous membranes

13. **Which vital sign is a priority for continuous monitoring to assess tissue perfusion in a patient with hemorrhagic shock?**
a) Respiratory rate
b) Mean arterial pressure (MAP)
c) Body temperature
d) Blood glucose level

14. **During monitoring of a patient in hemorrhagic shock, prolonged reduction in urine output may indicate:**
a) Improved renal perfusion
b) Heart failure
c) Renal hypoperfusion
d) Hypervolemia

15. **What is the target $SpO_2$ for a patient in hemorrhagic shock?**
a) 70-80%
b) 80-90%
c) $\geq$ 94%
d) 50-60%

**Table S2: General Self-Efficacy Scale**

| | Not at all true | Hardly true | Moderately true | Exactly true |
|---|---|---|---|---|
| 1. I can always manage to solve difficult problems if I try hard enough | ☐ | ☐ | ☐ | ☐ |
| 2. If someone opposes me, I can find the means and ways to get what I want. | ☐ | ☐ | ☐ | ☐ |
| 3. It is easy for me to stick to my aims and accomplish my goals. | ☐ | ☐ | ☐ | ☐ |
| 4. I am confident that I could deal efficiently with unexpected events. | ☐ | ☐ | ☐ | ☐ |
| 5. Thanks to my resourcefulness, I know how to handle unforeseen situations. | ☐ | ☐ | ☐ | ☐ |
| 6. I can solve most problems if I invest the necessary effort. | ☐ | ☐ | ☐ | ☐ |
| 7. I can remain calm when facing difficulties because I can rely on my coping abilities. | ☐ | ☐ | ☐ | ☐ |
| 8. When I am confronted with a problem, I can usually find several solutions. | ☐ | ☐ | ☐ | ☐ |
| 9. If I am in trouble, I can usually think of a solution | ☐ | ☐ | ☐ | ☐ |
| 10. I can usually handle whatever comes my way. | ☐ | ☐ | ☐ | ☐ |
